# Immunohistochemical Differentiation between Western and East Asian Types of CagA-Positive *Helicobacter pylori* in Gastric Biopsy Samples

**DOI:** 10.1155/2022/1371089

**Published:** 2022-11-12

**Authors:** Daisuke Kobayashi, Keisuke Uchida, Asuka Furukawa, Takashi Ito, Luis Masuo Maruta, Heinrich Bender Kohnert Seidler, Aloisio Felipe-Silva, Masaki Sekine, Noboru Ando, Yuka Toyama, Yusuke Chino, Keiko Miura, Kurara Yamamoto, Takumi Akashi, Yoshinobu Eishi, Kenichi Ohashi

**Affiliations:** ^1^Department of Human Pathology, Graduate School and Faculty of Medicine, Tokyo Medical and Dental University, Tokyo, Japan; ^2^Division of Surgical Pathology, Tokyo Medical and Dental University Hospital, Tokyo, Japan; ^3^Universidade de Sao Paulo (USP), Hospital Universitario, Endoscopy Service, Sao Paulo, SP, Brazil; ^4^Laboratório Brasiliense, Brasília, DF, Brazil; ^5^Universidade de Sao Paulo (USP), Hospital Universitario, Anatomic Pathology Service, Sao Paulo, SP, Brazil

## Abstract

**Background:**

Cag A-positive *Helicobacter pylori* isolated from human gastric mucosa is categorized as a Western or East Asian allele-type based on whether the *cagA* gene encodes an EPIYA-C or EPIYA-D motif. We aimed to differentiate between the 2 types of *H. pylori* by immunohistochemistry (IHC) using formalin-fixed paraffin-embedded (FFPE) gastric biopsy samples.

**Materials and Methods:**

We developed 2 monoclonal antibodies (mAbs) that detect either the EPIYA-C or EPIYA-D motif of the *H. pylori* CagA protein by IHC using FFPE tissues. FFPE tissue sections from 30 Japanese and 39 Brazilian gastric biopsy samples with *H. pylori* infection confirmed by Giemsa staining (moderate/severe in the Sydney classification system) were examined by IHC with the novel mAbs followed by polymerase chain reaction (PCR) for EPIYA-C or EPIYA-D using DNA extracted from adjacent tissue sections.

**Results:**

Differentiation among Western and East Asian types and CagA-negative *H. pylori* was successful in most (97%) samples by IHC with the novel mAbs and commercially available mAbs that react with a species-specific lipopolysaccharide or a common CagA motif of *H. pylori*. The detection status of EPIYA-C/D motifs by IHC with the novel mAbs was consistent with the PCR results in 61 (88%) of 69 samples: EPIYA-C(+)/D(−) in zero Japanese and 26 Brazilian samples, EPIYA-C(−)/D(+) in 26 Japanese and 1 Brazilian sample, and EPIYA-C(−)/D(−) in 1 Japanese and 7 Brazilian samples. The detection sensitivity and specificity of IHC with each novel mAb compared with the PCR results were, respectively, 84% and 97% for EPIYA-C, and 97% and 95% for EPIYA-D.

**Conclusions:**

The novel mAbs specific to each EPIYA-C or EPIYA-D motif differentiated between Western and East Asian types of CagA-positive *H. pylori* by IHC using FFPE tissues. Applying these novel mAbs to large numbers of archived pathology samples will contribute to elucidating the association of these allele types with gastric cancer.

## 1. Introduction

*Helicobacter pylori* infection is critically involved in the development of gastric cancer. [1] Although *H. pylori* infect almost half of the population worldwide, the incidence of gastric cancer does not necessarily coincide with the rate of *H. pylori* infection in individual countries. [2] One possible reason for this is the existence of pathogenic gene polymorphisms in *H. pylori* and their geographic differences [3].

The most important virulence factor of *H. pylori* is cytotoxin-associated gene A (CagA), and the gene encoding the CagA protein is located within the *cag* pathogenicity island (*cag* PAI). In CagA-positive or -negative strains of *H. pylori*, the *cag* PAI is either present or absent, respectively. [4] EPIYA motifs, characterized by the amino acid sequence Glu-Pro-lle-Tyr-Ala (EPIYA), locate at the C-terminal region of CagA protein. The 4 different types of EPIYA motifs, EPIYA-A, -B, -C, and -D, are determined by the surrounding amino acid sequence. The EPIYA-A and EPIYA-B motifs are representative polymorphisms shared by almost all *H. pylori* isolates, followed by the EPIYA-C or EPIYA-D motifs. [5] *H. pylori* strains with the EPIYA-C motif are referred to as the Western type and those with the EPIYA-D motif are referred to as the East Asian type according to their regional prevalence. [6] Compared with the EPIYA-C motif, the EPIYA-D motif binds more strongly to host SHP-2, an ubiquitously expressed protein tyrosine phosphatase recognized as a proto-oncogenic phosphatase [5, 7, 8]. Clinical observations suggest that infection with *H. pylori* carrying East Asian type CagA is associated with severe gastric atrophy and gastric cancer [9, 10].

The CagA status and genotype are mainly determined by polymerase chain reaction (PCR) and sequencing of the EPIYA region using DNA extracted from *H. pylori* isolated from fresh gastric biopsy samples. To gain more information on the CagA status in clinical and histopathologic studies of gastric cancer, we aimed to differentiate between the Western and East Asian types of CagA-positive *H. pylori* by immunohistochemistry (IHC) using formalin-fixed paraffin-embedded (FFPE) gastric biopsy samples. Toward this aim, we developed 2 monoclonal antibodies (mAbs) that detect either the EPIYA-C or EPIYA-D motif of the *H. pylori* CagA protein in FFPE tissue sections, and the sensitivity and specificity of IHC with the novel mAbs were compared with the PCR detection results for EPIYA-C or EPIYA-D using DNA extracted from adjacent FFPE tissue sections.

## 2. Materials and Methods

### 2.1. Tissue Samples

The FFPE tissue blocks of gastric biopsy samples obtained from 30 Japanese and 39 Brazilian patients infected with *H. pylori* (confirmed by Giemsa staining as moderate/severe in the Sydney classification system [11]) were collected from the Tokyo Medical and Dental University Hospital pathology archives in Japan, and the Sao Paulo University Hospital and Santa Cruz Hospital in Brazil. All patients underwent an endoscopic examination for dyspepsia or gastric cancer screening during the years 2020 and 2021, which revealed no evidence of malignancy endoscopically or pathologically. At least 1 tissue with *H. pylori* confirmed by Giemsa staining was included in the Japanese biopsy samples from the gastric antrum and/or corpus (different number of tissues) and in the Brazilian biopsy samples from the gastric antrum (3 tissues) and corpus (2 tissues). Serial tissue sections were cut from each FFPE tissue block of the gastric biopsy samples and subjected to IHC and PCR analysis. The study protocol received approval from the ethics committees of the Tokyo Medical and Dental University Hospital (reference M2018–244 and M2019–072) and the Sao Paulo University Hospital (reference HUUSP CEP76 CAAE 52744016.7.0000.0076).

### 2.2. mAb Production

Novel mAbs specific to either the EPIYA-C or EPIYA-D motif of *H. pylori* CagA protein were developed to determine the type (Western or East Asian) of *H. pylori* in FFPE gastric biopsy samples. The mAbs were generated according to a published laboratory protocol [12] with some modifications. BALB/c mice (CLEA Japan, Tokyo, Japan) were immunized with keyhole limpet hemocyanin-conjugated peptide synthesized by Eurofins Genomics (Ebersberg, Germany) from the whole sequence of EPIYA-C (N′-GFPLKRHDKVDDLSKVGRSVSPEPIYATIDDLGG-C′) or EPIYA-D (N′-AINRKIDRINKIASAGKGVGGFSGAGRSASPEPIYATIDFDEANQAG-C′). Hybridoma cell lines producing antibodies for each peptide were evaluated by enzyme-linked immunosorbent assay (ELISA) with the keyhole limpet hemocyanin-free synthetic peptide. Hybridomas with positive results were screened by manual IHC as described previously, [13] using FFPE gastric biopsy samples that were preliminarily confirmed by PCR to be infected with Western or East Asian type *H. pylori*. Finally, we selected the hybridoma that produced an antibody generating a strong reaction specific to each type of *H. pylori* on the gastric mucosa and cloned it by 2 rounds of limiting dilution. We implanted a single hybridoma clone in the intraperitoneal space in severe-combined immunodeficiency mice (CLEA Japan). Ascites was collected 1 or 2 weeks later and used without purification as an undiluted antibody. The antibodies obtained by immunization of either the EPIYA-C or EPIYA-D peptide antigen are referred to as EPIYA-C mAb (IgG2b,*κ*) or EPIYA-D mAb (IgG2b,*κ*), respectively, in the present study. The Institutional Animal Care and Use Committee of Tokyo Medical and Dental University approved all of the animal experiments (approval number: A2021-097A and approval date: 01/04/2021).

### 2.3. *H. pylori* Samples

The specificity of the novel EPIYA-C and EPIYA-D mAbs or the commercially available CagA mAb (sc-28368, Santa Cruz Biotechnology, CA, USA) specific to a common CagA motif of *H. pylori* was examined by ELISA and Western blotting with a standard strain of Western type *H. pylori* (ATCC 43504), [14] an isogenic *cagA*-knockout *H. pylori* mutant (ATCC 43504 Δ*cagA*) [14], and a clinical isolate of East Asian type *H. pylori* (HpLN-4) [13]. The *H. pylori* strains were cultured on sheep blood agar plates (BBL 252792, Tokyo, Japan) at 37°C in a jar with an AnaeroPack (Mitsubishi Gas Chemical Co., Inc., Tokyo, Japan) for 72 h. Whole cell lysates were prepared by sonicating the cultures on ice using an ultrasonic homogenizer (VP–5S ULTRA S homogenizer; Taitec, Koshigaya, Japan) at level 6 for 3 min with each bacterium in phosphate-buffered saline (PBS). The whole cell lysate was centrifuged at 12,000 rpm for 10 min, and the supernatant was collected and used for ELISA and Western blotting. A BCA protein assay kit (23225; Pierce, Rockford, IL) was used to quantify the protein in the supernatant.

### 2.4. ELISA

For the ELISA, flat-bottomed 96-well NUNC-immuno plates (Nalge Nunc International, Roskilde, Denmark) were coated with whole cell lysates of each of the 3 *H. pylori* strains (5 *µ*g per well) in carbonate-bicarbonate buffer (pH 9.6) for 90 min at 37°C. The mAbs were serially diluted in PBS containing 0.25% Tween-20 (T-PBS) and added to each well, and then, the plates were incubated for 90 min at 37°C. They were incubated further for 30 min with biotinylated rabbit anti-mouse immunoglobulins (E0354, DAKO, Glostrup, Denmark), followed by incubation for 30 min with horseradish peroxidase-conjugated streptavidin (P0397, DAKO), both at room temperature. The plates were washed with T-PBS both before and after each step. After the reaction, citrate phosphate buffer (pH 5.4) containing 0.3% o-phenylenediamine dihydrochloride (MilliporeSigma Co.) and 0.012% H_2_O_2_ was added to each well, and the plates were incubated in the dark for 15 min at room temperature. The reaction was stopped by adding 25 *µ*l of 2N HCl to each well. The plates were read at 490 nm on an ELx 808 TM Absorbance Microplate Reader (BioTek, Tokyo, Japan).

### 2.5. Western Blotting

Each whole cell lysate of the 3 *H. pylori* strains (12 *μ*g per lane) was separated by sodium dodecyl sulfate-polyacrylamide gel electrophoresis using 8% polyacrylamide gel. The samples were electrophoretically transferred to polyvinylidene difluoride membranes using a Mini Trans-Blot cell (Bio-Rad, Tokyo, Japan). Block Ace (DS Pharma Biomedical Co. Ltd., Osaka, Japan) was used to block the membranes overnight at 4°C and the membranes were then incubated with diluted antibodies (EPIYA-C mAb; 1 : 500, EPIYA-D mAb; 1 : 500, and CagA mAb; 1 : 500) at room temperature for 90 min. The membranes were incubated in the dark for 60 min with ECL Plex™ goat-*α*-mouse IgG-Cy3 (GE Healthcare, Buckinghamshire, UK) at a 1 : 1000 dilution ratio. The membranes were washed in T-PBS before and after each of the last 2 steps. Finally, the membranes were washed in PBS before being dried in the dark for 1 h at 37°C. A Bio-Rad BPC-500, Molecular Imager (Quantity One-Analysis Software Version 4.6.9, Bio-Rad) was used to image the dried membranes.

### 2.6. IHC

All samples were examined by IHC with the novel EPIYA-C and EPIYA-D mAbs and the commercially available CagA mAb and *H. pylori* mAb (D369-3, MBL, Nagoya, Japan) specific to *H. pylori* lipopolysaccharide (LPS). Serial tissue sections (4 *μ*m thick) mounted on silane-coated slides (Muto Pure Chemicals Co. Ltd., Tokyo, Japan) were subjected to automated IHC with the Leica BOND-III (Leica Microsystems Inc., Tokyo, Japan) using a BOND Polymer Refine Detection kit (#DS9800, Leica Microsystems Inc.). Deparaffinization, peroxidase inhibition, antigen retrieval, incubation with primary antibody at room temperature for 15 min, and counterstaining were performed according to the manufacturer's protocol. The best protocol for antigen retrieval was determined, as shown in Table 1. Appropriate dilution in Primary Antibody Diluent (10–0001RUO, Sakura Finetek Japan Co., Ltd., Tokyo, Japan) was determined for the *H. pylori* mAb; 1 : 1000, CagA mAb; 1 : 100, EPIYA-C mAb; 1 : 2000, and EPIYA-D mAb; 1 : 400. The IHC results were considered to be positive when at least 1 cluster of unequivocal dot-like signals was observed on the surface of the gastric mucosa in the biopsy sample.

### 2.7. PCR

A tissue section (10 *μ*m thick) from each biopsy sample was placed in a sterilized 1.5-ml centrifuge tube. The DNA of each section was extracted with TaKaRa DEXPAT™ (9091, Takara Shuzo, Shiga, Japan) and further purified by Ethachinmate (318–01793, Nippon Gene, Tokyo, Japan) according to the manufacturer's instructions. Real-time PCR was performed to amplify fragments of 16S ribosomal RNA, the *cagA* gene, and EPIYA-C and EPIYA-D, using primers and probes (supplementary Table 1) that were originally designed for droplet digital PCR for quantitative detection and genotyping of *H. pylori* from stool. [15,16] Real-time PCR mixtures contained 5 *μ*l of template DNA, 100-nM concentrations of each primer, a 40-nM concentration of the probe, and 2× SensiFAST Probe Hi-ROX Mix (BIO-82020, BIOLINE, Cincinnati, USA) in a total volume of 50 *μ*l. Amplification and detection were both performed using the ABI PRIZM 7900HT Sequence Detection System (Applied Biosystems, CA, USA) with the following program: 95°C for 5 min and then 50 cycles of 95°C for 15 s and 60°C for 1 min. Samples of serially diluted bacterial DNA were used as an internal standard to determine the amount of bacterial DNA in the sample used for PCR. The amount of bacterial DNA was expressed in terms of the number of bacterial genomes using 1.25 × 10^10^ Da per genome for the calculation [17]. Every PCR included negative controls without bacterial DNA. Positive samples were defined as those in which any bacterial genome was detected (supplementary Figure 1).

### 2.8. Statistical Analysis

Associations between the detection status of EPIYA-C or EPIYA-D by IHC and PCR were evaluated using Fisher's exact test. Unweighted Cohen's kappa coefficient was used to assess variability in the detection status of the EPIYA C/D motif by IHC and PCR. In the analysis, *p* values < 0.05 were considered statistically significant. All analyses were carried out with the statistical package R (version 3.6.3; available from http://www.r-project.org).

## 3. Results

### 3.1. Specificity of the Novel mAbs

**Figure 1:**
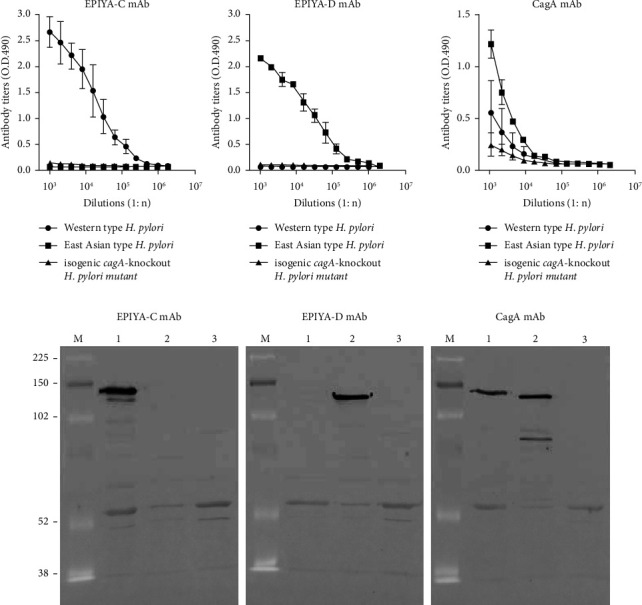
Specificity of the EPIYA-C and EPIYA-D mAbs by ELISA and Western blotting. The specificity of the novel mAbs or the CagA mAb was examined by ELISA (a) and Western blotting (b) using the sonicated whole cell lysate of a Western type *H. pylori* (ATCC 43504: lane 1), an East Asian type *H. pylori* (HpLN-4: lane 2), or an isogenic *cagA*-knockout *H. pylori* mutant (ATCC 43504 Δ*cagA*: lane 3). Among the 3 strains of *H. pylori*, the EPIYA-C mAb was specific to the Western type and the EPIYA-C mAb was specific to the East Asian type, while the CagA mAb reacted with both the Western and East Asian type CagA protein detected by the novel EPIYA-C or EPIYA-D mAb. Error bars indicate standard deviation of 3 identical wells. (M) Molecular size marker (kDa).

The novel EPIYA-C and EPIYA-D mAbs showed specific reactivity to each corresponding peptide without any cross-reactivity between them. In the ELISA and the Western blotting analysis using the sonicated *H. pylori* whole cell lysate, the EPIYA-C mAb reacted with the Western type *H. pylori* (ATCC 43504) but not with the East Asian type *H. pylori* (HpLN-4) or the *cagA*-knockout *H. pylori* (ATCC 43504 Δ*cagA*), whereas the EPIYA-D mAb reacted with the East Asian type *H. pylori* but not with the Western type *H. pylori* or the *cagA*-knockout *H. pylori* (Figure 1). The specificity of the CagA mAb was unclear in the ELISA but was confirmed by Western blotting, showing a sharp band corresponding to the Western type or East Asian type CagA protein detected by the EPIYA-C or EPIYA-D mAb, respectively.

### 3.2. *H. pylori* CagA Typing by IHC

**Figure 2:**
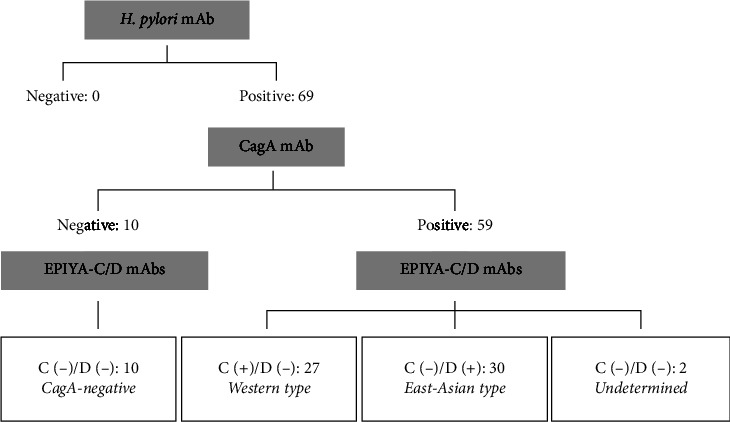
Algorithm for *H. pylori* CagA typing by IHC using gastric biopsy samples. The number of samples with positive or negative results by IHC with each of the *H*. *pylori* mAb (specific to species-specific LPS), the CagA mAb (specific to a common CagA motif), and the EPIYA-C/D mAbs (specific to either an EPIYA-C or EPIYA-D motif) are shown in the algorithm for CagA status determination. The detection status was positive (+) or negative (−) by IHC with the EPIYA-C mAb (C) or the EPIYA-D mAb (D). According to the algorithm, the CagA status of the 69 *H. pylori*-infected patients was determined as either the Western type, East Asian type, or CagA-negative *H. pylori*, excluding 2 patients with undetermined results.

**Figure 3:**
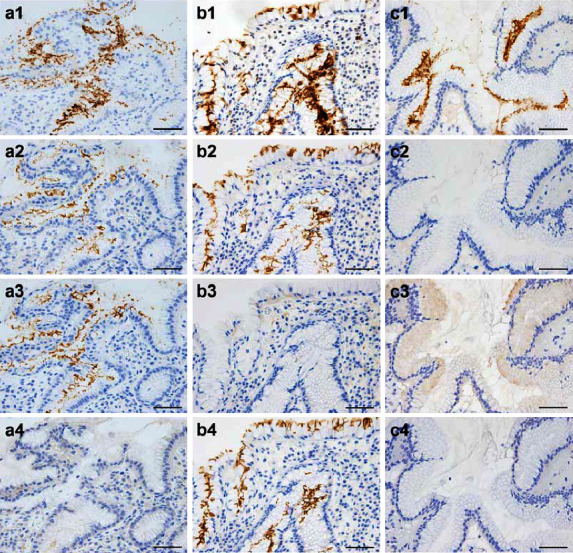
IHC features of *H. pylori* with different CagA status in the gastric mucosa. Representative pictures of IHC with the *H. pylori* mAb (1), the CagA mAb (2), the EPIYA-C mAb (3), and the EPIYA-D mAb (4) are shown pairwise for each identical gastric biopsy sample with the Western type (a), East Asian type (b), or CagA-negative (c) *H. pylori* infection confirmed by PCR afterward. The novel EPIYA-C and EPIYA-D mAbs showed a prominent contrast in the presence (a3/b4) or absence (a4/b3) of signals on the surface of gastric foveolar epithelium in the CagA-positive (a2/b2) samples with *H. pylori* infection (a1/b1). A sample with many *H. pylori* (c1) and minimum inflammation was CagA negative (c2) with no reactivity by the novel EPIYA-C/D mAbs (c3/c4). Bars: 50 µm.

The detection status of *H. pylori* by IHC with each antibody is shown in supplementary Table 2. By IHC, the *H. pylori* mAb reacted with Giemsa-positive *H. pylori* on the gastric mucosa in all 69 samples, and the CagA mAb reacted with some of the *H. pylori* mAb-positive *H. pylori* in 59 (86%) samples. Among the 59 CagA-positive samples, 27 samples (1 Japanese and 26 Brazilian patients) were positive for the EPIYA-C mAb and 30 samples (27 Japanese and 3 Brazilian patients) were positive for the EPIYA-D mAb; none of the samples was double positive for the 2 mAbs. The 2 positive and 10 negative samples by the CagA mAb were negative for both the EPIYA-C and EPIYA-D mAbs. According to the algorithm for CagA status determination (Figure 2), the *H. pylori* CagA typing by IHC with these mAbs was successful in 67 (97%) of the 69 gastric biopsy samples. Representative IHC pictures of *H. pylori* detected in gastric biopsy samples are shown, including the Western type; *H. pylori*(+)/CagA(+)/EPIYA-C(+)/EPIYA-D(−) (Figure 3(a)), the East Asian type; *H. pylori*(+) /CagA(+)/EPIYA-C(−)/EPIYA-D(+) (Figure 3(b)), and the CagA-negative *H. pylori*; *H. pylori*(+)/CagA(−)/EPIYA-C(−)/EPIYA-D(−) (Figure 3(c)). The CagA status was undetermined in the 2 (3%) samples that were positive for the *H. pylori* and CagA mAbs but negative for the EPIYA-C or EPIYA-D mAbs.

### 3.3. Correlation with PCR Detection Status

The number of *H. pylori* genomes detected by each PCR is shown in supplementary Table 2. By PCR, *H. pylori* 16S was detected in all 69 samples, including 57 (83%) samples with detection of *cagA*. The PCR detection status of both the EPIYA-C and EPIYA-D motifs was consistent with the IHC detection status in 61 (88%) samples (Table 2); EPIYA-C(+)/D(−) in zero Japanese and 26 Brazilian samples, EPIYA-C(−)/D(+) in 26 Japanese and 1 Brazilian sample, and EPIYA-C(−)/D(−) in the 1 Japanese and 7 Brazilian samples. The PCR results were inconsistent with the CagA status determined by IHC in 3 Japanese and 5 Brazilian samples. Statistical analysis revealed that the kappa coefficient for concordance of the EPIYA-C/D motif between the results obtained by IHC and PCR was 0.82 (*P* < 0.001). The PCR detection status of the EPIYA-C or EPIYA-D motif was consistent with the IHC detection status in 63 (91%) or 66 (96%) samples, respectively (Table 3). Compared with the PCR results, the IHC detection sensitivity and specificity were, respectively, 84% and 97% for EPIYA-C and 97% and 95% for EPIYA-D. The 2 samples with undetermined results by IHC were both positive by PCR for cagA; 1 from a Japanese patient was negative by PCR for both EPIYA-C and EPIYA-D and the other from a Brazilian patient was negative by PCR for EPIYA-D, but positive by PCR for EPIYA-C.

## 4. Discussion

For the present study, we developed novel mAbs that can detect either the EPIYA-C or EPIYA-D motif of CagA-positive *H. pylori* locating on the surface of FFPE gastric mucosa. Differentiation among the Western type, East Asian type, and CagA-negative *H. pylori* was successful in most (97%) of the gastric biopsy samples by IHC with the novel mAbs and commercially available mAbs that react with a species-specific LPS or a common CagA motif of *H. pylori*. The detection sensitivity and specificity of IHC with each novel mAb compared with the PCR results were, respectively, 84% and 97% for EPIYA-C, and 97% and 95% for EPIYA-D.

Validation of the *H. pylori* CagA typing by IHC was reported in 2020 by a research group from Oita University [18] who examined FFPE gastric biopsy samples by manual IHC with polyclonal antibodies (pAbs) raised against *H. pylori* (B0471, DAKO), CagA (b-300) (sc-25766, Santa Cruz, no longer available), or East Asian type CagA peptide (developed in their laboratory [19]). In their study, the pAb raised against East Asian type CagA peptide (N′-NAINRKIDRINKIASAGKG-C′) had markedly low sensitivity (23.9%) compared with the cagA sequence data of isolated *H. pylori* and was considered inappropriate for determining the CagA status. In the present study, we obtained novel mAbs for determining the CagA status by selecting and cloning the best hybridoma cell lines that produced a specific and strong reaction to the Western type or East Asian type of *H. pylori* on the gastric mucosa by IHC using FFPE tissue sections. Furthermore, an automated IHC method was used for the sample analysis to introduce the novel mAbs as a routine examination in pathology laboratories. Finally, unequivocal dot-like signals on the gastric mucosa with minimal background staining were obtained for Western or East Asian type *H. pylori* by the Leica system of IHC with the EPIYA-C or EPIYA-D mAbs, respectively.

The commercially available *H. pylori* mAb (D369-3, MBL) that reacts with a species-specific LPS was originally produced in our laboratory (clone: TMDU-D8) by immunizing mice with sonicated whole bacterial lysate of the Western type *H. pylori* followed by screening hybridoma cell lines by IHC using FFPE tissue sections of the *H. pylori*-infected stomach resected from a Japanese gastric cancer patient.[13] The *H. pylori* mAb was confirmed to be highly specific for *H. pylori* species by ELISA and Western blotting, and the IHC detection sensitivities for *H. pylori* in the gastric mucosa and gastric lymph nodes were higher than those of the commercially available anti-*H. pylori* pAb (DAKO).[13] In the present study, the *H. pylori* mAb strongly reacted with *H.* pylori in all gastric biopsy samples from Japanese and Brazilian patients with *H. pylori* infection confirmed by Giemsa staining, indicating that *H. pylori* expresses high levels of species-specific LPS within the cell wall outer membrane structure regardless of the CagA status. This antibody specific to *H. pylori* LPS was useful for detection of both CagA-negative and CagA-positive *H. pylori*.

The commercially available CagA mAb (Santa Cruz sc-28368) was raised against a common CagA motif comprising amino acids 1–300 of CagA of *H. pylori* origin, according to the manufacturer's information. In the Western blotting analysis performed in the study, the CagA mAb reacted with both the Western type and East Asian type *H. pylori*, whereas the novel EPIYA-C or EPIYA-D mAb specifically reacted with either the Western type or East Asian type *H. pylori*, respectively. The detection status by IHC with the CagA mAb was almost consistent with the results by IHC with the novel EPIYA-C/D mAbs; IHC with the CagA mAb was positive in all 57 samples with either EPIYA-C or EPIYA-D detected, and negative in the 10 samples with both EPIYA-C and EPIYA-D undetected. According to the algorithm for determining the CagA status, we could differentiate among Western type, East Asian type, and CagA-negative *H. pylori* in 67 (97%) of the 69 gastric biopsy samples. The 3 Brazilian samples with EPIYA-D detected by IHC were all from patients of Japanese descent, and the 1 Japanese sample with EPIYA-C detected by IHC was from a foreign resident in Japan (private communications). Although it is not clear why the 2 (3%) samples were positive by the CagA mAb but negative by both the EPIYA-C/D mAbs, the possibility of a CagA-positive *H. pylori* without an EPIYA-C/D motif, such as a unique CagA type called ABB-type CagA, [18] cannot be ruled out, at least in the case of the Japanese sample with consistent results obtained by IHC and PCR.

To estimate the accuracy of *H. pylori* CagA typing by IHC with the novel mAbs, we performed PCR typing for the EPIYA-C/D motif using total tissue DNA extracted from FFPE gastric biopsy samples because *H. pylori* isolates from fresh biopsy samples were not obtained from these patients. While the detection status of the EPIYA-C/D motif was consistent between the results by IHC and PCR in many (88%) samples, we suspect that the inconsistent results in the 8 (12%) samples were likely due to the use of DNA extracted from FFPE tissue sections for the PCR. Formalin fixation usually causes DNA fragmentation, cytosine deamination, and cross-linking [20] and decreases PCR sensitivity. [21] Contamination by *H. pylori* carrying the prevalent type CagA into the tissue from other samples with *H. pylori* infection may occur during tissue processing for paraffin-embedding in identical chambers of the machine used in pathology laboratories. [22–24] Locating CagA-positive *H. pylori* carrying either Western or East Asian type CagA by IHC on the gastric mucosa surface can exclude the possibility of such unexpected contamination between FFPE tissue samples prepared for conventional pathologic examination. Further studies to compare the IHC results with the genotyping results of *H. pylori* isolates are needed to determine the reasons for the inconsistent results obtained by IHC and PCR in some samples in this study.

The currently available *H. pylori* genotyping methods for assessing the presence of known virulence genes or alleles require the use of DNA extracted from *H. pylori* isolated by culturing fresh gastric biopsy samples. Because of such restrictions, information about *H. pylori* CagA status is not easily available for routine pathologic examination. Successful *H. pylori* CagA typing by automated IHC with the novel mAbs using FFPE tissue samples enables diagnosis of the CagA status of *H. pylori*-infected patients by conventional histopathologic examination and would thus be useful for examining how the gastric pathologies differ between patients infected with Western type or East Asian type of *H. pylori*. In the present study, we did not compare the histopathology as the Western type or East Asian type of *H. pylori* infection was detected in most Brazilian or Japanese patients, respectively. Further studies comparing clinical outcomes and gastric pathologies between patients infected with a different *H. pylori* CagA status using IHC with the novel mAbs are expected, especially in countries where the 2 types of *H. pylori* are more or less prevalent in their populations such as in Thailand [25–27] and Okinawa, Japan [10, 28, 29].

In conclusion, the novel mAbs specific to each EPIYA-C or EPIYA-D motif could differentiate between Western and East Asian types of CagA-positive *H. pylori* by automated IHC using FFPE tissue sections. Application of these novel mAbs to large numbers of samples accumulated in pathology archives will contribute to elucidating the potential associations of these *H. pylori* allele types with gastric cancer.

## Figures and Tables

**Table 1:** Monoclonal antibodies and antigen retrieval protocol used for the Leica IHC detection system.

Antibodies	Company and catalog number	Specific to	Antigen retrieval (buffer, temperature, Duration)
*H. pylori* mAb	MBL, C369-3	*H. pylori* LPS	ER2, 100°C, 20 min
CagA mAb	SCB, sc-28368	*H. pylori* CagA	ER2, 100°C, 20 min
EPIYA-C mAb	–	EPIYA-C motif	ER1, 100°C, 20 min
EPIYA-D mAb	–	EPIYA-D motif	ER2, 100°C, 20 min

IHC: immunohistochemistry, mAb: monoclonal antibody, LPS: lipopolysaccharide. MBL : Medical and Biological Laboratories, Tokyo, Japan, SCB : Santa Cruz Biotechnology, CA, USA, ER2, ER1: antigen retrieval with BOND Epitope Retrieval Solution 2 or Solution 1 (#AR9960 or #AR9961, Leica Microsystems Inc., Tokyo, Japan).

**Table 2:** Detection status of both EPIYA-C and EPIYA-D motifs of *H. pylori* in gastric biopsy samples by IHC and PCR.

Detection status of both EPIYA-C/D motifs by IHC	Number of samples with the IHC result	Number of samples with detection of both EPIYA-C/D motifs by PCR			
		C (+)/D (−)	C (−)/D (+)	C (−)/D (−)	C (+)/D (+)
C (+)/D (−)	27	26^*∗*^	0	1	0
C (−)/D (+)	30	1	27^*∗*^	1	1
C (−)/D (−)	12	3	1	8^*∗*^	0
C (+)/D (+)	0	0	0	0	0

Detection by IHC or PCR was positive (+) or negative (−) for the EPIYA-C (C) or EPIYA-D (D) motif. IHC: immunohistochemistry, ^*∗*^Concordance rate of the EPIYA-C/D motif was 88% (Κappa = 0.82, *P* < 0.001 by unweighted Cohen's kappa coefficient).

**Table 3:** Detection sensitivity and specificity of IHC for an EPIYA-C or EPIYA-D motif of *H. pylori* in gastric biopsy samples compared with the PCR results.

Detection status of each motif By IHC	Number of samples with the PCR results		*P* value^*∗*^	Sensitivity (%)	Specificity (%)
	PCR (+)	PCR (−)			
*EPIYA-C motif*					
IHC (+)	26	1	<0.001	84	97
IHC (−)	5	37			

*EPIYA-D motif*					
IHC (+)	28	2	<0.001	97	95
IHC (−)	1	38			

Detection by IHC or PCR was positive (+) or negative (−) for EPIYA-C or EPIYA-D motif. IHC: immunohistochemistry, ^*∗*^Fisher's Exact Test.

## Data Availability

The underlying data supporting the results of this study are listed at Supplementary Materials section and attached as supplementary files.

## Abbreviations

CagA: Cytotoxin-associated gene A
ELISA: Enzyme-linked immunosorbent assay
FFPE: Formalin-fixed paraffin-embedded
IHC: Immunohistochemistry
LPS: Lipopolysaccharide
mAb: monoclonal antibody
pAb: polyclonal antibody
PAI: pathogenicity island
PCR: polymerase chain reaction
SHP-2: SRC homology 2 domain-containing tyrosine phosphatase.

## Supplementary Materials

Supplementary Materials Supplementary Figure 1: representative amplification curves of the internal standard and the positive or negative samples detected by real-time PCR. Supplementary Table 1: sequences of primers and probes used for real-time PCR. Supplementary Table 2: detection status of *H. pylori* in all samples by IHC with each antibody and by PCR for each gene.

## References

[1] Uemura N., Okamoto S., Yamamoto S. (2001). *Helicobacter pylori* infection and the development of gastric cancer. *New England Journal of Medicine*.

[2] Amieva M. R., El-Omar E. M. (2008). Host-bacterial interactions in *Helicobacter pylori* infection. *Gastroenterology*.

[3] Chang W. L., Yeh Y. C., Sheu B. S. (2018). The impacts of *H. pylori* virulence factors on the development of gastroduodenal diseases. *Journal of Biomedical Science*.

[4] Censini S., Lange C., Xiang Z. (1996). *cag*, a pathogenicity island of *Helicobacter pylori*, encodes type I-specific and disease-associated virulence factors. *Proceedings of the National Academy of Sciences of the United States of America*.

[5] Higashi H., Tsutsumi R., Fujita A. Biological activity of the *Helicobacter pylori* virulence factor CagA is determined by variation in the tyrosine phosphorylation sites.

[6] Azuma T. (2004). *Helicobacter pylori* CagA protein variation associated with gastric cancer in Asia. *Journal of Gastroenterology*.

[7] Chan R. J., Feng G. S. (2007). PTPN11 is the first identified proto-oncogene that encodes a tyrosine phosphatase. *Blood*.

[8] Higashi H., Tsutsumi R., Muto S. (2002). SHP-2 tyrosine phosphatase as an intracellular target of *Helicobacter pylori* CagA protein. *Science*.

[9] Azuma T., Yamazaki S., Yamakawa A. (2004). Association between diversity in the Src homology 2 domain--containing tyrosine phosphatase binding site of *Helicobacter pylori* CagA protein and gastric atrophy and cancer. *The Journal of Infectious Diseases*.

[10] Satomi S., Yamakawa A., Matsunaga S. (2006). Relationship between the diversity of the cagA gene of *Helicobacter pylori* and gastric cancer in Okinawa, Japan. *Journal of Gastroenterology*.

[11] Dixon M. F., Genta R. M., Yardley J. H., Correa P. (1996). Classification and grading of gastritis. The updated sydney system. international workshop on the Histolopathology of gastritis, Houston 1994. *American Journal of Surgical Pathology*.

[12] Harlow E. L., Lane D. (1988). Antibodies: a laboratory manual. *Cold Spring Harbour*.

[13] Ito T., Kobayashi D., Uchida K. (2008). *Helicobacter pylori* invades the gastric mucosa and translocates to the gastric lymph nodes. *Laboratory Investigation*.

[14] Wada Y., Takemura K., Tummala P. (2018). *Helicobacter pylori* induces somatic mutations in *TP53* via overexpression of CHAC1 in infected gastric epithelial cells. *FEBS Open Bio*.

[15] Talarico S., Safaeian M., Gonzalez P. (2016). Quantitative detection and genotyping of *Helicobacter pylori* from stool using droplet digital PCR reveals variation in bacterial loads that correlates with cagA virulence gene carriage. *Helicobacter*.

[16] Talarico S., Leverich C. K., Wei B. (2018). Increased *H. pylori* stool shedding and EPIYA-D cagA alleles are associated with gastric cancer in an East Asian hospital. *PLoS One*.

[17] Kobayashi D., Eishi Y., Ohkusa T. (2002). Gastric mucosal density of *Helicobacter pylori* estimated by real-time PCR compared with results of urea breath test and histological grading. *Journal of Medical Microbiology*.

[18] Miftahussurur M., Doohan D., Syam A. F. (2020). The validation of the *Helicobacter pylori* CagA typing by immunohistochemistry: nationwide application in Indonesia. *Acta Histochemica*.

[19] Uchida T., Kanada R., Tsukamoto Y. (2007). Immunohistochemical diagnosis of the cagA-gene genotype of *Helicobacter pylori* with anti-East Asian CagA-specific antibody. *Cancer Science*.

[20] Do H., Dobrovic A. (2015). Sequence artifacts in DNA from formalin-fixed tissues: causes and strategies for minimization. *Clinical Chemistry*.

[21] Mielonen O. I., Pratas D., Hedman K., Sajantila A., Perdomo M. F. (2022). Detection of low-copy human virus DNA upon prolonged formalin fixation. *Viruses*.

[22] Hockney R., Orr C. H., Waring G. J. (2022). Formalin-Fixed Paraffin-Embedded (FFPE) samples are not a beneficial replacement for frozen tissues in fetal membrane microbiota research. *PLoS One*.

[23] Lam S. Y., Ioannou A., Konstanti P. (2021). Technical challenges regarding the use of formalin-fixed paraffin embedded (FFPE) tissue specimens for the detection of bacterial alterations in colorectal cancer. *BMC Microbiology*.

[24] Mena M., Lloveras B., Tous S. (2017). Development and validation of a protocol for optimizing the use of paraffin blocks in molecular epidemiological studies: the example from the HPV-AHEAD study. *PLoS One*.

[25] Subsomwong P., Miftahussurur M., Vilaichone R. K. (2017). *Helicobacter pylori* virulence genes of minor ethnic groups in North Thailand. *Gut Pathogens*.

[26] Chomvarin C., Phusri K., Sawadpanich K. (2012). Prevalence of cagA EPIYA motifs in *Helicobacter pylori* among dyspeptic patients in northeast Thailand. *Southeast Asian Journal of Tropical Medicine and Public Health*.

[27] Boonyanugomol W., Kongkasame W., Palittapongarnpim P. (2020). Genetic variation in the cag pathogenicity island of *Helicobacter pylori* strains detected from gastroduodenal patients in Thailand. *Brazilian Journal of Microbiology*.

[28] Duncan S. S., Valk P. L., Shaffer C. L., Bordenstein S. R., Cover T. L. (2012). J-Western forms of *Helicobacter pylori* cagA constitute a distinct phylogenetic group with a widespread geographic distribution. *Journal of Bacteriology*.

[29] Matsunari O., Shiota S., Suzuki R. (2012). Association between *Helicobacter pylori* virulence factors and gastroduodenal diseases in Okinawa, Japan. *Journal of Clinical Microbiology*.

