# Supplementary material for: Immunohistochemical Differentiation between Western and East Asian Types of CagA-Positive Helicobacter pylori in Gastric Biopsy Samples
Source: Can J Gastroenterol Hepatol. 2022 Nov 12;2022:1371089. doi: 10.1155/2022/1371089 (PMC9678484; doi:10.1155/2022/1371089)
Supplement: Supplementary Materials — Supplementary Figure 1: representative amplification curves of the internal standard and the positive or negative samples detected by real-time PCR. Supplementary Table 1: sequences of primers and probes used for real-time PCR. Supplementary Table 2: detection status of H. pylori in all samples by IHC with each antibody and by PCR for each gene. [file 1371089.f1.zip › Supplementary Figure 1 (1).docx]

**Supplementary Figure 1**: Representative amplification curves of the internal standard and the positive or negative samples detected by real-time PCR

Internal standard


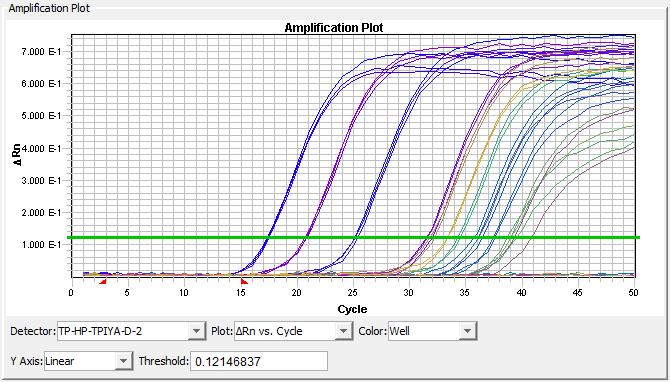


**positive**

**negative**
