# Supplementary material for: Immunohistochemical Differentiation between Western and East Asian Types of CagA-Positive Helicobacter pylori in Gastric Biopsy Samples
Source: Can J Gastroenterol Hepatol. 2022 Nov 12;2022:1371089. doi: 10.1155/2022/1371089 (PMC9678484; doi:10.1155/2022/1371089)
Supplement: Supplementary Materials — Supplementary Figure 1: representative amplification curves of the internal standard and the positive or negative samples detected by real-time PCR. Supplementary Table 1: sequences of primers and probes used for real-time PCR. Supplementary Table 2: detection status of H. pylori in all samples by IHC with each antibody and by PCR for each gene. [file 1371089.f1.zip › Supplementary Table 1 (2).docx]

**Supplementary Table 1**: Primers and probes used for real-time PCR

| *H. pylori* gene | Sequences |
| --- | --- |
| 16S rRNA | Forward : 5’– GCGACCTGCTGGAACATTAC – 3’  Reverse : 5’ – CGTTAGCTGCATTACTGGAGA – 3’  Probe : 5’ HEX – AAGCCCTCCAACAACTAGCATCCAT – BHQ1 3’ |
| CagA | Forward : 5’ – TGGCTCAAGCTCGTGAAT – 3’  Reverse : 5’ – TGGAAAACTTGAACGAATCAGA – 3’  Probe : 5’ FAM – CTTCCYACATTATGYGCAACKATC – BHQ1 3’ |
| EPIYA-C | Forward : 5’ – TCAGTTAGCCCTGAACC – 3’  Reverse : 5’ – GCCCTACCTTACTGAGAT – 3’  Probe : 5’ HEX – GAAAGCCCTACTTTACTGAG – BHQ1 3’ |
| EPIYA-D | Forward : 5’ – TCAACTAGCCCTGAACC – 3’  Reverse : 5’ – GAAAGCCCTACTTTACTGAG – 3’  Probe : 5’ FAM – AAGCCTGCTTGATTTGCCTCATCAAA – BHQ1 3’ |

FAM: Carboxyfluorescein, BHQ1: Black Hole Quencher 1.
